# First report of Crimean-Congo hemorrhagic fever virus exposure in human and livestock populations, Center Region, Cameroon

**DOI:** 10.3389/fcimb.2025.1578518

**Published:** 2025-06-09

**Authors:** Morena Gasparine, Armand Namekong Fokeng, Eva Lopez, Stephanie Mvodo, Laurence Thirion, Archile Paguem, Remi Charrel, Xavier de Lamballerie, Alessandra Falchi

**Affiliations:** ^1^ Emerging Viruses Unit (UVE: University of Aix-Marseille, University of Corsica, Research Institute for Development (IRD) 190, National Institute of Health and Medical Research (Inserm) 1207, Armed Forces Biomedical Research Institute (IRBA)), Corte, France; ^2^ Faculty of Agriculture and Veterinary Medicine, University of Buea, Buea, Cameroon; ^3^ University of Aix-Marseille, National Reference Centre for Arboviruses, Marseille, France

**Keywords:** CCHFV, cattle, humans, goats, ticks, Cameroon, one health, Akonolinga

## Abstract

Crimean-Congo hemorrhagic fever (CCHF) is a widespread, tick-borne viral infection present in many African countries. Its epidemiology and impact on public health remain poorly understood in Cameroon. The main objective of the current study was to investigate the circulation of CCHF virus (CCHFV) in ruminants (cattle and goats), humans, and ticks collected simultaneously in a study area of Akonolinga, a health district in the central region of Cameroon. From the 15 to 28 July 2024, a cross-sectional study was conducted in Akonolinga, by collecting survey data and serum samples (from humans, goats, and cattle) and picking ticks from cattle and goats. This study included 100 randomly selected households from eight localities. Data were also collected using questionnaires to assess CCHFV seropositivity-associated factors. Individual characteristics of 189 goats and 246 cattle were collected and the data geo-referenced. To assess the prevalence of CCHF, serological enzyme-linked immunosorbent assay (ELISA) and molecular (real-time Reverse Transcriptase (RT) PCR) methods were used to detect antibodies targeting CCHF viral nucleoprotein and CCHFV-specific RNA in collected sera, respectively. The presence of CCHFV-specific RNA was also assessed in tick homogenate using real-time RT PCR. The overall CCHFV seroprevalence was 1.9% [95% CI (1.02%–3.64%)] in humans, 10.9% [n = 42; 95% CI (8.15%–14.38%)] in cattle, and 3.38% [n = 5; 95% CI (1.45%–7.66%)] in goats. Seroprevalence in cattle increased significantly with age. A total of 554 ticks were collected from 162 of the 386 (42%) cattle examined, with *Rhipicephalus* (*Boophilus*) *microplus* being the dominant species. CCHFV RNA was detected in two sera of women sampled. Phylogenetic analysis of a small portion of the L segment classified the strain within the African genotype III. This study reported, for the first time, the proven exposure of the human population to CCHFV in central Cameroon, showing strong evidence that CCHFV is infecting humans. Serological analyses revealed exposure of cattle and goats to CCHFV-strains collected in the same geographical area. These results demonstrate the potential risk of CCHF emergence in the human population, especially in rural areas in close vicinity with animals.

## 1 Introduction

Crimean-Congo hemorrhagic fever (CCHF) is caused by a tick-borne virus belonging to the genus *Nairovirus* within the family *Bunyaviridae* (Abudurexiti et al., 2019). Since its identification, CCHF has been recognized as a major public health threat (Formenty et al., 2007) as the most prevalent tick-borne human hemorrhagic fever virus with case fatality rates of 5%–30% and a large geographic distribution (including Africa, Asia, Eastern and Western Europe, and the Middle East) (Formenty et al., 2007). In Western Europe, the virus has notably been reported in Spain where both autochthonous human cases and viral circulation in ticks have been documented (Negredo et al., 2017; Sánchez-Seco et al., 2022). The primary vectors for CCHF virus (CCHFV) transmission are ticks of the genus *Hyalomma*, although some species from other genera such as *Rhipicephalus* show experimental evidence of competence (Gargili et al., 2017a). Ticks can be infected vertically (i.e., from parent to offspring) or horizontally by co-feeding with infected ticks or on a viremic animal (Spengler et al., 2016a). Wild mammals (mainly ungulates, lagomorphs, and rodents) and domestic animals (such as cattle, sheep, and goats) are hosts for immature and adult ticks and are, therefore, important in the ecology of CCHFV (Al-Abri et al., 2017). Viremia in domestic and infected animals can last from 2 to 15 days, and species that experience prolonged viremia (e.g., some lagomorphs) might act as amplifying hosts. In humans, primary infection occurs after an infected tick bite, by accidental crushing of infected ticks or through direct contact with tissues, fluids, or blood of viremic hosts (Spengler et al., 2016b). Household contacts of infected patients and healthcare personnel are at risk for exposure through contact with patient blood and secretions or needlesticks (Leblebicioglu et al., 2016).

There is a limited understanding of the epidemiology of CCHF, particularly in Sub-Saharan Africa, where the absence of clinical cases or infrequent acute case detections often contrasts with seroprevalence data, indicating substantial exposure in humans and livestock. Cameroon is an ecologically diverse country in Central-Western Sub-Saharan Africa characterized by five agroecological zones (Yengoh and Ardö, 2014). The actual epidemiology knowledge of CCHFV in Cameroon is poorly documented and mainly based on seroprevalence values observed on pastoral cattle originating from the Northern regions with values up to 98% in cattle from Adamawa, North and Far North regions (Simo et al., 2024). To date, a portion of the L segment of CCHFV (445 nt) has been detected in pools of *Hyalomma truncatum* collected from cattle of Northern regions and characterized as belonging to genotype III (Simo Tchetgna et al., 2023). Data about the exposure of the human population to CCHFV are scarce, and, to our knowledge, the virus has never been detected in humans in Cameroon. Seroprevalence rates for CCHFV in the human populations ranged from 0.5% (Balinandi et al., 2021) in Northern Cameroon to 4.4% among Pygmies in the eastern region (Sadeuh-Mba et al., 2018) to 17.8% in cattle herders originating from the western region of Cameroon (Simo et al., 2024).

Thus, the epidemiological knowledge of CCHFV in Cameroon is mainly based on epidemiological data of livestock originating from the Sudano-Sahelian agro-ecological zone characterized by i) dry savannah and steppes; ii) the presence of several species of *Hyalomma* genus (Marie Thérèse et al., 2021), and iii) a high density of cattle population estimated at 3.55 million heads (MINEPIA, 2013). In contrast, the Center Region of Cameroon, is characterized by a humid forest-savanna mosaic with a bi-modal rainfall, an important frequency of detection of *Rh.* (*Boophilus*) *microplus* (Awa et al., 2015; Kiwan et al., 2025b) and a sporadic detection of ticks belonging to the genus *Hyalomma* (Sado Yousseu et al., 2022). Those observations can be explained by the fact that *Rh.* (*Boophilus*) *microplus* is an invasive tick species found across four of the five agro-ecological zones of Cameroon, indicating its broad distribution (Silatsa et al., 2019). In contrast, the population of *Hyalomma* ticks is generally low, although they are present all year-round (Silatsa et al., 2019). As a two-host tick, its dynamics are closely tied to the presence of intermediate hosts. This can also be attributed to the biology and environmental conditions of the ticks. Specifically, *Hyalomma* spp. are known to prefer warm and semi-arid environments, whereas *Rhipicephalus* spp. are more commonly found in warm and humid conditions (Ayalew et al., 2014). Thus, in the Center Region, CCHF ecology might differ from elsewhere and can provide new information on the epidemiology of CCHFV in a different eco-epidemiological context with respect to Northern Cameroon. Given the role of vectors, animals (wild and domestic fauna), and humans in the maintenance of CCHFV, a comprehensive understanding requires a One Health approach. In the present study, we investigated, for the first time, the occurrence of CCHFV in large (cattle) and small (goats) ruminants, humans, and ticks simultaneously in Akonolinga, a health district located in the Center Region, Cameroon.

## 2 Methodology

### 2.1 Ethics

This study was approved by the research ethics review committee (Ref number: 00104/CRERSHC/2024) and by the Ministry of Scientific Research and Innovation (MINRESI) (Ref number: 000097/MINRESI/B00/C00/C10/C13). An informed written consent was obtained from adults (or parents/legal guardians in the case of children under 20 years of age). Oral consent for blood and tick sampling was obtained from the animal’s owners.

### 2.2 Study area

**Figure 1:**
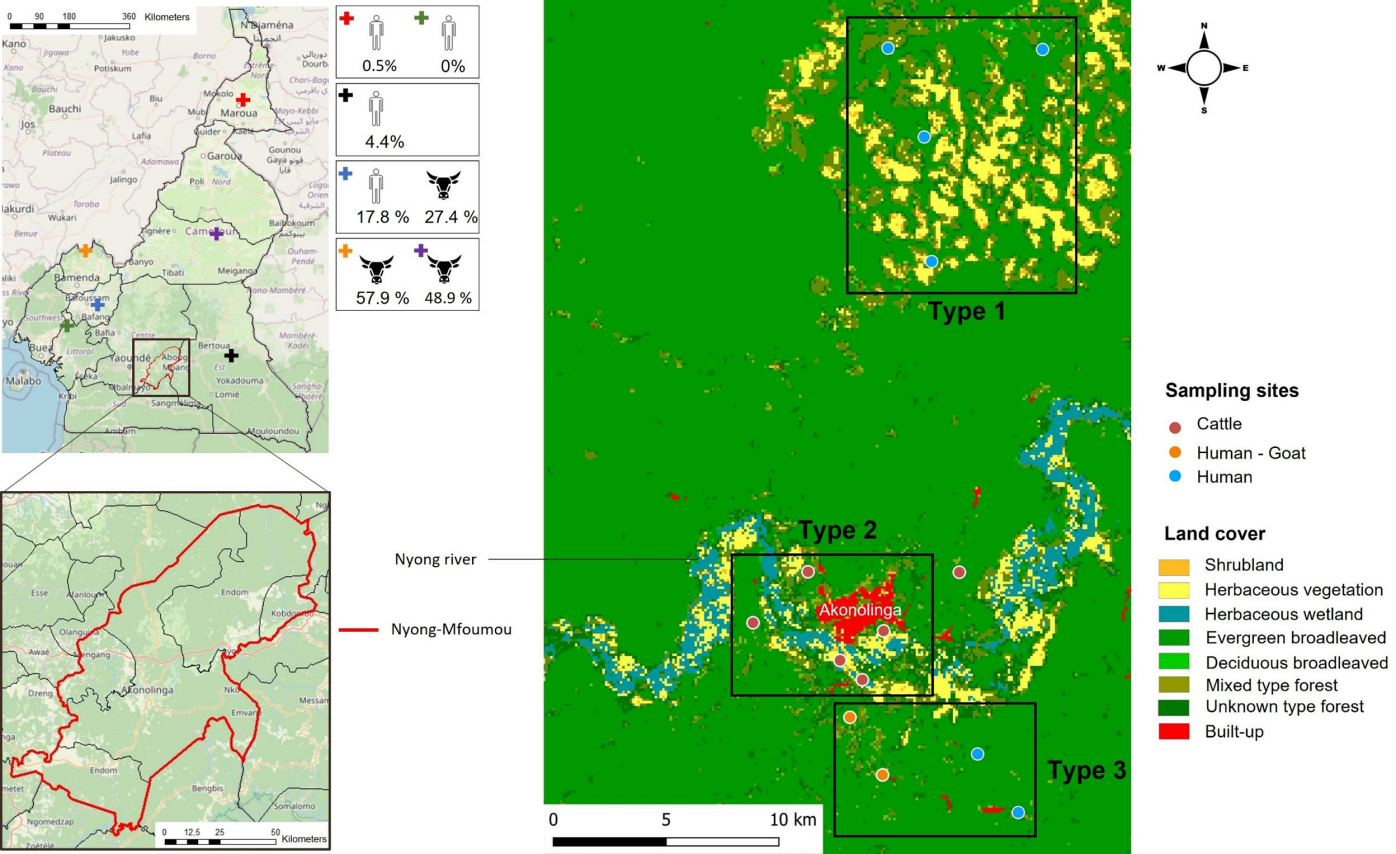
Map of the Nyong-Mfoumou department depicting the study region; the sites of sampling for humans, goats, and cattle; and the land cover associated. Districts where previous studies were conducted among humans and cattle in Cameroon (Gonzalez et al., 1989; Sadeuh-Mba et al., 2018; González Gordon et al., 2022; Simo et al., 2024) are indicated by a cross.

The Akonolinga health district is located in the Nyong-et-Mfoumou Division, 100 km east from Yaoundé, Center Region of Cameroon, with a population of about 105,789 inhabitants in 2015 (**Figure 1**). According to the Koppen classification, the Akonolinga district climate is considered as an Equatorial savannah (Aw code) (Hufty, 2001) with temperatures above 20°C throughout the year, high annual rainfall, and no winter season. The environmental conditions are influenced by seasonality, as this zone has a sub-tropical climate with two rainy seasons per year (August to November and April to May) and two dry seasons (December to March and July to August) (Molua, 2006).

The vegetation cover of the entire survey area was identified using a raster file from Copernicus Global Land Service (Land Cover 2019 (Raster 100 m), Global, Yearly – Version 3, 2025). Overall, the ecosystem is characterized by an evergreen broadleaved forest. The study area (**Figure 1**) has been grouped into three different types of vegetation cover. The first type, where only human populations have been sampled, was characterized by a dominant presence of savannah (herbaceous and shrubby vegetation). The second type, where only cattle were sampled, was very close to the center of Akonolinga and runs alongside the Nyong River. It differs from type 1 by the presence of herbaceous wetland. Finally, the third type, where humans and goats have been enrolled, was characterized by broadleaved and mixed evergreen forest (**Figure 1**).

The Nyong River basin in this area, which has several tributaries throughout the villages, is characteristically known for its swampy banks, which is the contact point between human population and domestic and wild animals, also providing suitable habitats for several arthropods including vectors of pathogen (Ndengué et al., 2019). The populations are typically rural, and their main activities are farming, fishing, and subsistence livestock rearing (mainly poultry, goats, sheep, and pigs) within close range of human living quarters. In this area, cattle and sheep are raised by the Fulani ethnic group, a pastoral community spanning Central and West Africa (Bruijn et al., 2016). The Fulani tribe graze mainly Zebu *White fulani* cattle on extensive communal pastures. Some herders still practice transhumance (seasonal migration) in the dry season along Nyong River valleys in search of pasture as shown in **Figure 1**. The cattle population in the Center Region of Cameroon is estimated at 276,855 heads. In Akonolinga, the cattle population is estimated at 6675 heads and sheep population at 1250 heads, reared under an open grazing system along the Nyong River mainly (unpublished data communicated by Ministère de l'Élevage, des Pêches et des Industries Animales (MINEPIA)). In the study area, in contrast to cattle and sheep, goats are usually reared by the local population around the house and generally kept in small numbers not exceeding three goats per rural household.

### 2.3 Study population, design, and setting

Between 15 and 28 July 2024, a cross-sectional study was conducted in Akonolinga (**Figure 1**), collecting survey data and serum samples from humans, goats, and cattle and picking ticks from the cattle and goats.

#### 2.3.1 Human population

As small ruminants have been recognized as CCHFV hosts in certain endemic regions and have been epidemiologically linked to human cases, we selected seven villages on the basis of the presence of small ruminants (on average, each village included had more than 50% of owners of small ruminants). The seven villages, comparable in terms of size (100–150 households each), accounted for a population of at least 1,500 individuals. The target sample size for human participants was 462 individuals to give ±2% precision on prevalence estimates with a 5% confidence level, assuming 4% of individuals would be positive for Immunoglobulin G (IgG) and accounting for 20% of sample failure. We planned to enroll 100 households and for each household, up to five individuals. Then, within each cluster (village), as the list of households was not available, household-level selection has been performed using random-walk method, which has been used in earlier studies to ensure that surveyors advance in a random direction and select random houses along the way (Lemeshow and Robinson, 1985). A household was defined as a person or group of persons, whether related or not, who live together in the same dwelling, who recognize an adult man or woman as the head of the household, who share the same household arrangements, and who are considered as a single unit. Once a household was selected, five members aged 5 years and above were selected randomly and asked to enroll in the study.

#### 2.3.2 Livestock

We obtained permission to sample goats in three of the eight settlements in which humans were included. For logistical purposes, the goats sampled had been moved to a common location to facilitate sampling, so it was not possible to associate the goats to their households. Six cattle herds within a maximum radius of 10 km of the village survey area were included in the study (**Figure 1**). The targeted livestock herds were relatively stable in their location and not directly associated with commercial livestock trade networks. The sample size of ruminants was estimated at 211 for goats (to give ±2% precision on prevalence estimates with a 5% confidence level, assuming 2% of individuals would be positive for IgG) and at 428 for cattle (to give ±3% precision on prevalence estimates with a 5% confidence level, assuming 10% of individuals would be positive for IgG).

### 2.4 Data collection

#### 2.4.1 Human population

A door-to-door survey was carried out by two mobile teams of three individuals each. The teams were led by a local community health worker with previous experience of community-based health campaigns (e.g., vaccination or mosquito nets distribution). The data collectors were one Cameroonian nurse and two French epidemiologists.

For each included household, the head of household or his spouse was invited to respond to a structured questionnaire developed with KoboKollect^©^, a free software for epidemiological survey (Lakshminarasimhappa, 2022). Questionnaire was administered through face-to-face interviews in French or Ewondo languages to make sure that all participants understood the questions. Data collected about respondents included their gender, age, occupation, education level, household size, number of children aged less than 5 years old, presence of domestic animals and pets, exposure to animal blood or tissues, tick-bite frequency, onset of symptoms after tick bites, and other socio-behavioral activities such as crushing ticks with their bare hands. The other four members of the household included in the study reported their age and sex, which were recorded using a questionnaire that was related to that of the head of the household. Immediately after the interview, the investigator asked for permission to collect a blood sample from each of the five participants. The blood was collected by the nurse in a 5-mL sterile Vacutainer tube with coagulation activator and serum separator. All participants underwent a rapid diagnostic test (RDT) for malaria [First Response^®^ Malaria Antigen *P. falciparum* (HRP2) Card Test] (First Response® Malaria Antigen P.Falciparum (HRP2) Card Test, 2025).

#### 2.4.2 Livestock

Collected information for each sampled animal (goats and cattle) was gender, age, GPS/location, ticks, and a blood sample. Veterinarians of the MINEPIA were engaged for tick and blood collection from each selected animal. At each site, all the cattle and goats available at the time of the inspection were examined for the presence of ticks (adults and immature stages). Animals were restrained and kept standing and all the body parts of the cattle were examined. Ticks were collected using blunt steel forceps and placed inside corning centrifuge tubes. In parallel, blood was collected in a 5-mL sterile Vacutainer tube with coagulation activator and serum separator from the jugular veins. We identified ticks by using taxonomic keys and then pooled ticks by species, sex, development stage, study site, and animal host, as previously reported (Walker et al., 2003).

### 2.5 Laboratory analysis

#### 2.5.1 CCHFV antibody detection

As CCHFV is a biosafety level–4 pathogen that should be handled in high confinement settings, often not available or affordable by each institution (Weidmann et al., 2016), human and livestock serum samples were subjected to heat inactivation at 56°C for 2 h (Donets et al., 1977) prior to shipment for serological and molecular analyses at Unité des Virus Emergents (France). At least 200 µL of serum was obtained from each sample after clotting by centrifugation at 1,000–2,000 × g for 10 min. Serum samples were tested in duplicate following the manufacturers’ instructions using Innovative diagnostic (ID) screen CCHF double antigen multi-species ELISA kits (IDVet, Grabels, France) according to the manufacturer’s instructions. Briefly, 96-well microtiter plates were precoated with recombinant CCHFV nucleoprotein antigen. Samples with sample–to–positive control ratio (S/P %) greater than 30% were determined positive.

#### 2.5.2 Nucleic acid extraction

##### 2.5.2.1 Ticks

Ticks, whether individual or grouped, were homogenized in 1 mL of Minimal Essential Medium solution (containing antibiotics and fungicide) using the TissueLyser II (QIAGEN, Hilden, Germany) at a frequency of 30 cycles per second for 3 min. Before nucleic acid extraction, a standardized quantity of MS2 bacteriophages (10 µL per 100 µL of homogenate) was added to each sample to monitor the steps of nucleic acid extraction, reverse transcription, and amplification by RT-qPCR (European Virus Archive Global (EVAg), 2020). Nucleic acids were extracted from 200 µL of tick homogenate using magnetic beads with the KingFisher Flex™ system and the MagMAX™ Viral/Pathogen Ultra Nucleic Acid Isolation Kit (Thermo Fisher Scientific), following the manufacturer’s instructions. After extraction, the nucleic acids were suspended in 100 µL of the elution buffer and stored at −80°C for long-term preservation.

##### 2.5.2.2 Humans

Nucleic acids from human samples were extracted using the QIAamp Viral RNA Mini Kit and QIAcube HT Plasticware (QIAGEN), following the manufacturer’s protocol. The extracted nucleic acids were then suspended in 90 µL of the elution buffer and stored at −80°C for further analyses. All samples were inactivated prior molecular biological analyses using buffer VXL.

#### 2.5.3 Genome detection of CCHFV and sequencing

Ticks’ homogenates and human and animal sera were tested for CCHF virus RNA using RT-qPCR systems targeting the large (L) RNA segment as previously described (Balinandi et al., 2021). RT-qPCR amplicon sequencing was performed to validate the sample’s positive status. The effective detection of CCHFV genome was strongly supported by the formal exclusion of contamination because the PCR systems used can distinguish genomic RNA from the positive control (Ninove et al., 2011). Amplicons were purified with the Monarch PCR & DNA Cleanup Kit (5 µg) (New England Biolabs), following the manufacturer’s instructions, and analyzed using the Oxford Nanopore Technologies’ MinION Mk1B sequencer paired with the SQK NBD114.24 kit (New England Biolabs) following the manufacturer’s instructions.

### 2.6 Sequence alignments and phylogeny analysis

In addition to the CCHFV sequences obtained, 59 other sequences available on the NCBI Virus platform (accessible at https://www.ncbi.nlm.nih.gov/labs/virus/vssi/#/) were included in the alignments for phylogenetic analyses. The sequences were aligned using ClustalW, and a scaled phylogenetic tree was constructed using the maximum likelihood method with the Kimura two-parameter distance, utilizing the Mega11 software (MEGA11: Molecular Evolutionary Genetics Analysis Version 11 | Molecular Biology and Evolution | Oxford Academic, 2025).

### 2.7 Statistical analysis

Sociodemographic, epidemiological, and laboratory data were summarized using frequencies and percentages. Village-level and herd-level seroprevalences were calculated by determining the percentage of positives/total number samples with binomial confidence intervals. Factors associated with CCHFV seroprevalences were evaluated by bivariate and multivariate logistic regression analyses. Variables for the model were chosen through automatic backward selection using Akaike information criterion. Results were presented as odds ratio with 95% confidence intervals [OR (95% CI)]. A 95% confidence interval and a significance level of 5% were used to determine statistical significance of the multivariate model. Village-level seroprevalence for humans, cattle, and goats was plotted on maps of the study area with Quantum GIS (QGIS) version 3.16.0. Moran’s I was calculated using *spdep package* in R. A Moran’s statistic of 1 is equivalent to perfect spatial clustering, whereas a value of −1 represents perfect dispersal. All analyses were performed using the R-4.4.2 program.

## 3 Results

**Figure 2:**
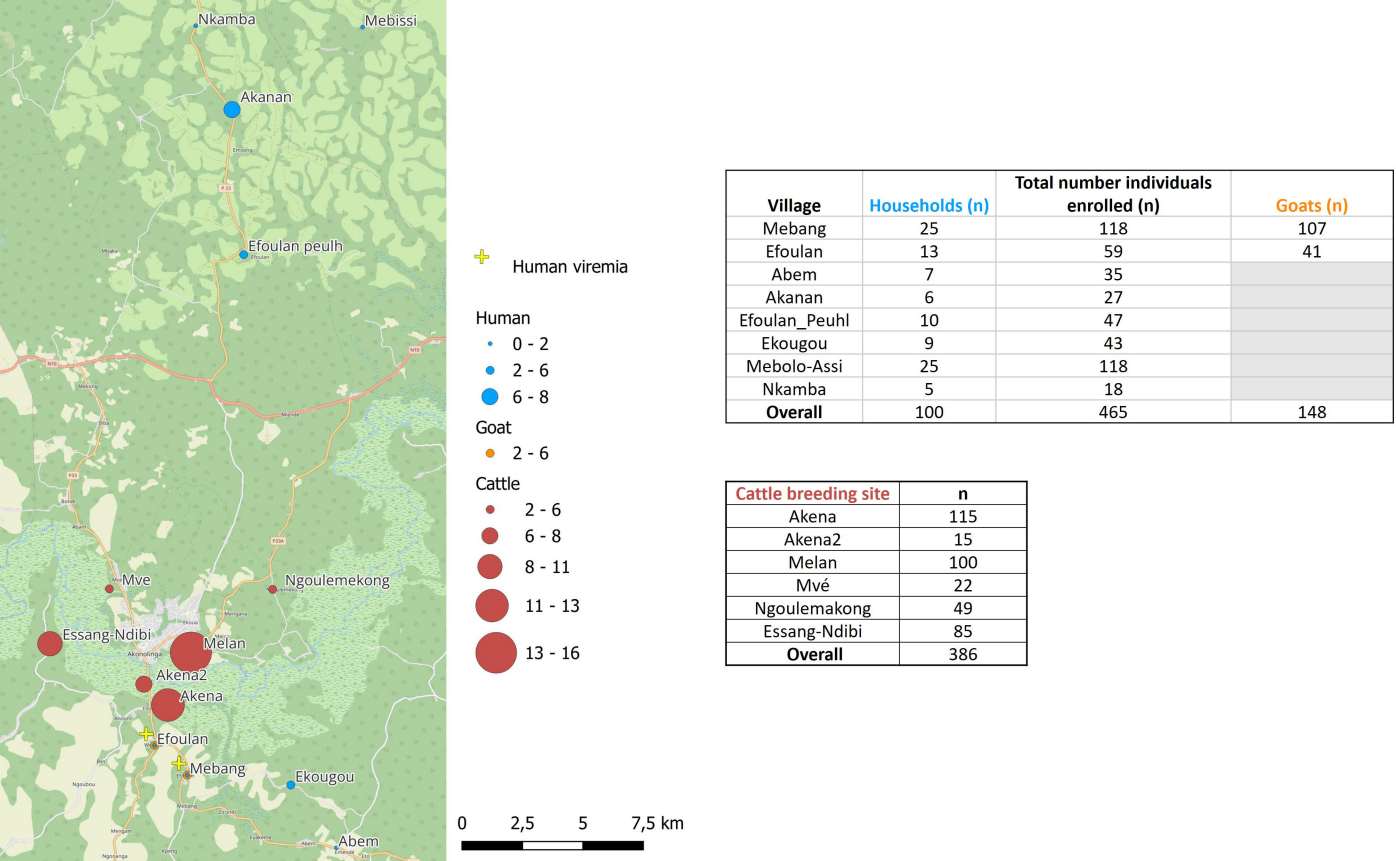
Map of the study area showing the seroprevalence rates and viremia detection for Crimean-Congo hemorrhagic fever virus in the studied sites for human, cattle, and goats.

The study obtained a total of 465, 148, and 386 samples from humans, goats, and cattle, respectively (**Figure 2**).

### 3.1 Baseline characteristics of the human population

We approached and successfully completed interviews at 100 households in which 465 individuals have been included from eight settlements (**Figure 2**): 7 households from Abem, 6 from Akanan, 13 from Efoulan, 10 from Efoulan Peuhl, 9 from Ekougou; 25 from Mebang; 25 from Mebolo Assi, 5 from Nkamba (**Figure 2**). **Table 1** summarizes the main characteristics of the household’s respondents surveyed. Of the 100 household respondents, 72% were men and had a mean age of 50 (min = 21 and max = 79). Agriculture was the most common activity carried out by respondents, with 94 individuals. Half of respondents had attained a secondary level of education. The household size was >10 for 48% of households. Ninety eight percent of households had domestic animals, of which 59% had at least a goat and 61% had a dog. CCHFV risk factors explored are shown in **Supplementary Table S1** (annex). Ninety one percent of respondents declared to slaughter animals in their households, 93% declared to know ticks, 33% declared had history of tick bites on time in life, and 27% during the last 12 months. They declared to crush ticks with bare hands for 69% of them, and 3% have ever eaten engorged ticks in their life. Four respondents declared to have developed symptoms after a tick bite, and they declared fever and headaches. Among the 100 household respondents, 41% showed positivity to malaria RDT, and five showed anti-CCHFV IgG antibodies.

**Table 1:** Socio-demographic characteristics of household respondents.

Characteristics of household respondents	Overall
	N = 100*^1^*
Gender	
Male	72 (72%)
**Age n (%); Moy Min–Max**	50 (21, 79)
Age groups (years)	
21–45	31 (31%)
46–60	30 (30%)
≥61	28 (28%)
**Head of household (yes)**	89 (89%)
Study level	
Illiterate	16 (16%)
Primary	30 (30%)
Secondary	52 (52%)
Tertiary	2 (2.0%)
Activities	
Farming	94 (94%)
Other	6 (6%)
Size household	
1–5	20 (20%)
6–10	32 (32%)
>10	48 (48%)
**Domestic animals (yes)**	97 (98%)
*Pigs*	44 (46%)
*Poultry*	89 (93%)
*Goats*	57 (59%)
**Pets (yes)**	77 (77%)
*Dog (yes)*	47 (61%)
**CCHFV seropositivity**	5 (5%)
**Malaria RTD positivity**	41 (41%)

*^1^* n (%).

### 3.2 CCHFV-specific antibody seroprevalence in humans and livestock

Among the 465 individuals enrolled from the 100 households, 53% (n = 247) were women, and the mean age was 29 years (5–88) (**Table 2**). Overall, CCHFV human seroprevalence was of 1.9% [n = 9; 95% CI (1.02%–3.64%)], with values ranging from 1.69% [95% CI (0.47%–5.97%)] in Mebolo Assi and Mebang to 7.4% [95% CI (2.06%–23.37%)] in Akanan (**Figure 2**; **Supplementary Table S2**). The nine individuals showing seropositivity to CCHFV had a mean age of 42 years (5–76), and 78% (n = 7) were men (**Supplementary Table S3**). They belonged to nine distinct households from five of the eight settlements investigated (**Figure 2**). There was no statistical difference in age (p = 0.08) and sex (p = 0.09) between those who were seropositive and those who were seronegative with respect to CCHFV. However, among seropositive participants, there is a significant difference between men and women (p = 0.017). Five of the nine CCHFV-seropositive individuals have responded to risk factors survey as household respondents. They had a mean age of 58 years old (35–76), and they were all men and were originating from five distinct households from Mebang (n = 1), Akenan (n = 2), Efoulan (Peuhl) (n = 1), and Ekougou (n = 1). All declared to slaughter animals in their households and to know ticks. Forty percent (n = 2) were declared to have a history of tick bites on time in life and 20% (n = 1) during the last 12 months. They also responded to crush tick with bare hands for 80% of them. Anyone of the seropositive participants declared to have developed symptoms after a tick bite.

**Table 2:** Characteristics of all individuals enrolled from the 100 households.

Sociodemographic characteristics	Anti-CCHFV antibodies		Overall	p-value*^2^*
	Negative	Positive		
	N = 456*^1^*	N = 9*^1^*	N = 465*^1^*	
**Age n (%); Moy Min–Max**	29 (5–88)	42 (5–76)	29 (5–88)	
**Age group (years)**				0.08
5–14	166 (36%)	2 (22%)	168 (36%)	
15–29	104 (23%)	1 (11%)	105 (23%)	
30–44	64 (14%)	2 (22%)	66 (14%)	
45–65	88 (19%)	1 (11%)	89 (19%)	
≥65	34 (7.5%)	3 (33%)	37 (8.0%)	
**Gender**				0.09
Female	245 (54%)	2 (22%)	247 (53%)	
Male	211 (46%)	7 (78%)	218 (47%)	
**Village**				0.3
Abem	35 (7.7%)	0 (0%)	35 (7.5%)	
Akanan	25 (5.5%)	2 (22%)	27 (5.8%)	
Efoulan	59 (13%)	0 (0%)	59 (13%)	
Efoulan Peuhl	46 (10%)	1 (11%)	47 (10%)	
Ekougou	41 (9.0%)	2 (22%)	43 (9.2%)	
Mebang	116 (25%)	2 (22%)	118 (25%)	
Mebolo Assi	116 (25%)	2 (22%)	118 (25%)	
Nkamba	18 (3.9%)	0 (0%)	18 (3.9%)	
**Malaria Rapid Diagnostic test**				0.9
Positive	208 (46%)	5 (56%)	213 (46%)	
Negative	218 (48%)	4 (44%)	222 (48%)	
Unknown	30 (6.6%)	0 (0%)	30 (6.5%)	

*^1^* n (%).

*^2^* Fisher’s exact test.

*^1^* n (%); Moy, Mean (min–max, Min–Max).

We investigated six cattle herds (**Figure 2**; **Table 3**). Among 386 cattle, 71% (n = 273) were females, and 68% (n = 260) were aged between 1 and 5 years: 10.9% [n = 42; 95% CI (8.15%–14.38%)] were positive for anti-CCHFV antibody with values ranging from 2.04% [95% CI (0.36%–10.69%)] in Ngoulemakong to 12.17% [95% CI (7.39%–19.40%)] in Akena. All six herds had at least one seropositive animal (**Figure 2**). Cattle showing seropositivity (n = 42) were significantly older than those showing seronegativity (n = 344; p < 0.001). The odds of being seropositive increased with age (p < 0.001) and was 3.3 [95% CI (1.6–6.2)] times higher in cattle of 6–10 years old compared to 6–12 months old in adjusted OR (p = 0.002).

**Table 3:** Demographic characteristics and CCHFV seroprevalence in cattle investigated.

Characteristics	All cattle	Seronegativity	Seropositivity	p-value*^2^*	Univariate analysis Multivariable analysis					
	N = 386*^1^*	N = 344*^1^*	N = 42*^1^*		OR*^1^*	95% CI*^1^*	p-value	Adjusted OR*^1^*	95% CI*^1^*	p-value
Village										
Ngoulemakong	49 (13%)	48 (14%)	1 (2.4%)	0.090	Ref	—				
Mve	22 (5.7%)	21 (6.1%)	1 (2.4%)		2.29	0.09, 59.7	0.6			
Akena2	15 (3.9%)	14 (4.1%)	1 (2.4%)		3.43	0.13, 90.5	0.4			
Essang-Ndibi	85 (22%)	77 (22%)	8 (19%)		4.99	0.88, 94.0	0.14			
Akena	115 (30%)	101 (29%)	14 (33%)		6.65	1.28, 122	0.071			
Melan	100 (26%)	83 (24%)	17 (40%)		9.83	1.92, 180	0.029			
Animal sex										
Male	113 (29%)	105 (31%)	8 (19%)	0.12	Ref	—				
Female	273 (71%)	239 (69%)	34 (81%)		1.87	0.88, 4.46	0.13			
Age groups (year)										
6–12 months	62 (16%)	61 (18%)	1 (2.4%)	<0.001	Ref	—		—	—	
1–2 years	142 (37%)	135 (39%)	7 (17%)		3.16	0.55, 59.8	0.3	1.1	−0.69, 4.0	0.3
3–5 years	118 (31%)	103 (30%)	15 (36%)		8.88	1.73, 163	0.037	2.2	0.54, 5.1	0.037
6–10 years	64 (17%)	45 (13%)	19 (45%)		**25.8**	**5.05, 471**	**0.002**	**3.3**	**1.6, 6.2**	**0.002**
Tick collected										
No	224 (58%)	202 (59%)	22 (52%)	0.4	Ref	—		—	—	
Yes	162 (42%)	142 (41%)	20 (48%)		1.29	0.68, 2.46	0.4			

*^1^* n (%).

*^2^* Pearson’s Chi-squared test.

Overall, we included 148 goats (**Figure 2**), of which 78% (n = 116) were females and 68% were older than 1 year. The global seroprevalence was 3.38% [n = 5; 95% CI (1.45%–7.66%)]. Four of the five goats showing seropositivity were sampled in Mebang village, where exposure to CCHFV was also observed in two individuals, and one goat showing seropositivity was sampled in Efoulan village. Seroprevalence rates were higher in cattle than in goats (p = 0.0124). Assessment of spatial autocorrelation via Moran I statistic showed no evidence of spatial autocorrelation in human and livestock populations (**Table 4**).

**Table 4:** Seroprevalence of CCHFV in human and livestock populations, in Akonolinga area, Cameroon.

			Seroprevalence range per village/breed (95% CI)			
Species	No. tested	Overall seroprevalence	Low	High	Moran *I*	p-value
Cattle	386	10.39 (7.72–13.84)	2.04 (0.36–10.69)	16 (10.0–24.42)	−0.22	0.72
Goats	148	3.33 (1.4–7.6)	2.44 (0.43–12.60)	3.74 (1.46–9.22)	−0.64	0.19
Humans	465	1.9 (1.02–3.64)	2.13 (0.38–11.11)	7.4 (2.06–23.37)	−0.5	0.28

### 3.3 Tick species and infection rates

A total of 554 ticks, organized in 235 pools containing an average of 2 ticks (range, 1–10 ticks), were collected from 162 of the 386 cattle examined. Overall, tick infestation rate was 42% [95% CI (37.15%–46.95%)]. The rate of tick infestation varied significantly across cattle breeding sites (p < 0.001). Female cattle were significantly more infested than male cattle (p = 0.0033) (**Table 5**). Among the 554 ticks, 446 were females and 98 were males. Ten were nymphs. They consisted of *Rhipicephalus* (*B*) *microplus* (472; 85.20%), *Amblyomma variegatum* (81; 14.62%), and one *Rhipicephalus* spp. (1; 0.18%). Goats enrolled in this study were not infested by ticks.

**Table 5:** Tick infestation rates by main characteristics of cattle.

Cattle characteristics	Not infested by ticks	Infested by ticks	p-value*^2^*
	N = 224*^1^*	N = 162*^1^*	
**Breed site**			<0.001
Akena	54 (24%)	61 (38%)	
Akena2	14 (6.3%)	1 (0.6%)	
Essang-ndibi	42 (19%)	43 (27%)	
Melan	73 (33%)	27 (17%)	
Mve	15 (6.7%)	7 (4.3%)	
Ngoulemakong	26 (12%)	23 (14%)	
**Sex**			0.033
Female	149 (67%)	124 (77%)	
Male	75 (33%)	38 (23%)	
**Age groups**			0.4
6–12 months	42 (19%)	20 (12%)	
1–2 years	79 (35%)	63 (39%)	
3–5 years	68 (30%)	50 (31%)	
6–9 years	35 (16%)	29 (18%)	
**CCHFV IgG**	22 (9.8%)	20 (12%)	0.4
**Overall tick infestation rates**	42% (37.15%–46.95%)		

*^1^* n (%).

*^2^* Pearson’s Chi-squared test.

### 3.4 CCHFV detection in ticks, livestock, and humans

**Figure 3:**
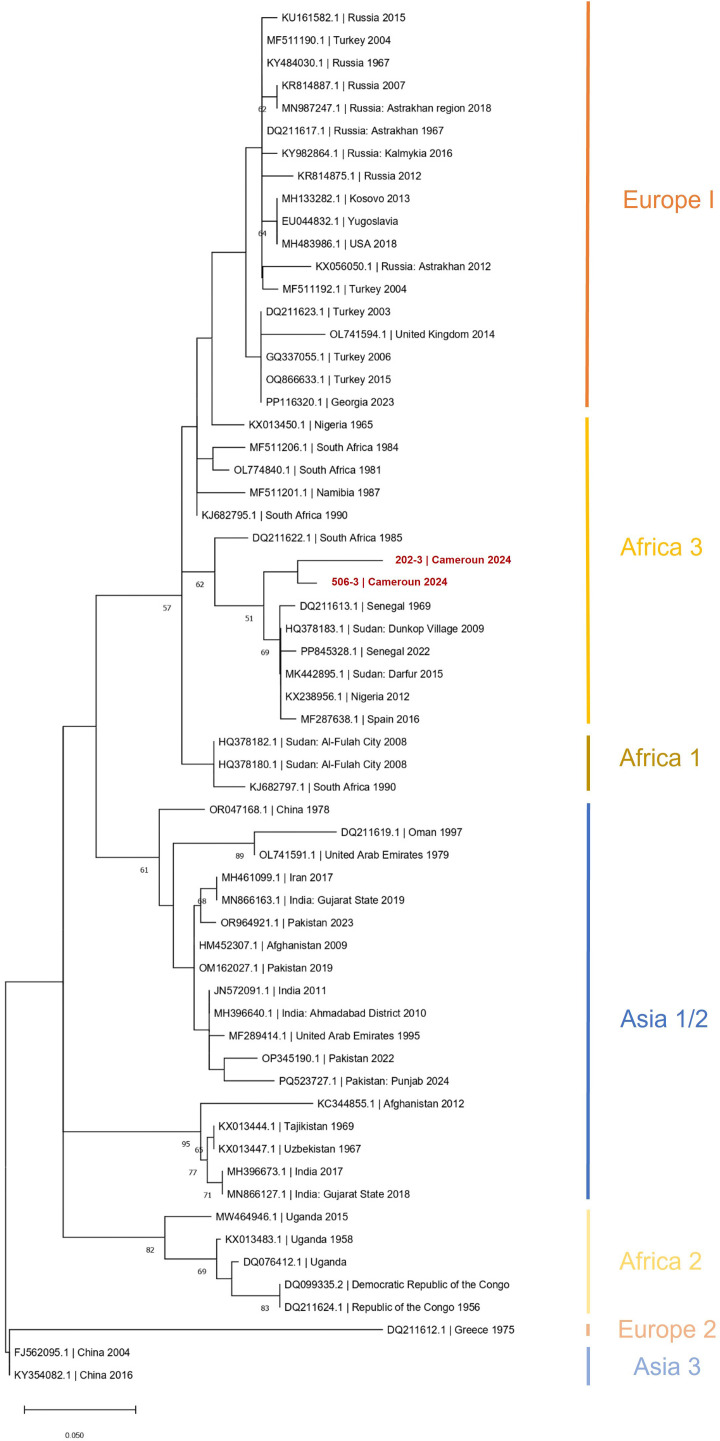
Phylogenetic tree based on 61 partial (150 nt) sequences of the CCHFV L segment. Tree was constructed using the maximum-likelihood method. Results of the bootstrap test (500 replicates) are shown next to the branches. Genotypes are indicated by Roman numerals with the equivalent clade nomenclature: Africa I, West Africa (Africa 1); II, Central Africa (Africa 2); III, South and West Africa (Africa 3); IV, Middle East/Asia, divided into two groups Asia 1 and Asia 2; V (Europe 1), Europe/Turkey (Europe 1); VI, Greece (Europe 2); VII (Europe 3). Scale bars indicate nucleotide substitutions per site. CCHFV, Crimean-Congo hemorrhagic fever virus; L-RNA, large segment of CCHFV RNA.

CCHFV-RNA was detected neither in ticks nor in animal sera. Among the 465 human sera, two were positive with the CCHFV-L RT-qPCR assay (Ct = 37.79 and Ct = 42.62), which was confirmed by amplicon sequencing. These two individuals were both women, aged 13 and 50 years old, respectively. They did not show antibodies against CCHFV and were negative to malaria RDT. They were sampled from Mebang and Efoulan villages, respectively (**Figures 1**, **2**). In Mebang, the seroprevalence was 1.69% [95% CI (0.47%–5.97%)] in humans and 3.7% [95% CI (1.46%–9.22%)] in goats. In Efoulan village, no human had CCHFV antibodies, whereas 1/41 goat had CCHFV antibodies (**Figure 2**). Phylogenetically, the two CCHFV sequences grouped with other sequences corresponding to the genotype III (also known as Africa 3) (**Figure 3**); these two sequences were most closely related with other human sequences identified in Nigeria, Senegal, Sudan, and Spain.

## 4 Discussion

This study presents the first confirmed evidence of human exposure to the CCHFV in Cameroon while analyzing exposure in cattle, goats, and ticks collected from the same rural area of the center region of Cameroon. In the rural human population of Akonolinga, the overall seroprevalence of CCHF IgG antibodies is consistent with rates reported in North (0.5%; 2/395) (Gonzalez et al., 1989) and East (4.4%; 6/137) (Sadeuh-Mba et al., 2018) Cameroon. However, it is significantly lower than the 17.8% (5/33) reported in herders and the 8.3% (5/65) reported in febrile patients from West Cameroon (Simo et al., 2024). This suggest a lower exposure to CCHFV in Akonolinga despite our population had several risk factors such as farming, outdoor activities, and managing domestic animals (slaughterhouses activities and crushing ticks with bare hands). The reported abattoir activities were mainly resulting in contacts with poultry, goats, and pigs, which limits the risk of CCHFV exposure compared to cattle-related abattoir (Simo Tchetgna et al., 2023). This study also revealed the presence of CCHFV RNA in 0.4% of tested human sera (2 positive human sera out of 465 tested). Indeed, both individuals who tested positive did not have symptom, which aligns with reports most of CCHFV infections (>80%), are asymptomatic or mild (Belobo et al., 2021). This is showing circulation of CCHFV in Mebang and Efoulan displaying geographic proximity and similar vegetation type (a broadleaved and mixed evergreen forest). We did not detect CCHFV antibodies in the two viremic human samples. This may be explained by the finding that viremia usually resolves around the time anti-CCHFV antibodies develop (Sahak et al., 2019). The fact that the two individuals were a woman of 50 years old and a girl of 13 years old is in line with a previous study in Afghanistan showing that almost half of the CCHFV cases were reported among 16–30 years old and that, for the occupation, most of those reported were housewives (15%). Housewives might be at risk because of being in contact with the blood of animals while butchering and cooking and not using protection methods (Kaya et al., 2014). Goats sampled in these two villages also showed exposure to CCHFV, especially in the site of Mebang. Altogether, the data collected in humans and goats strengthen the hypothesis that the virus is circulating in this area.

Phylogenetic analyses based on a small region of the L segment showed that the virus strain belongs to the African III genotype. Although this conclusion is based on sequence data from only one of the three genome segments, it aligns with a similar analysis of a CCHFV detected in ticks collected from cattle in Northern Cameroon (Simo Tchetgna et al., 2023). Most phylogenetic analyses for CCHFV genotyping are typically based on the S segment. However, some studies have shown that the genotypes derived from both the S and L segments are generally consistent, reflecting the geographic distribution of the virus (Bente et al., 2013). In Central Africa, CCHFV exhibits high genetic diversity, with the African II and III genotypes being particularly prominent (Grard et al., 2011). Nonetheless, given the considerable genetic recombination and reassortment, which can complicate genotype classification, these findings should be considered preliminary. The absence of S and M segment data limits a comprehensive understanding of CCHFV evolution. The S segment provides strong phylogenetic resolution due to its sequencing availability, whereas the M segment offers insights into potential differences in virulence and host adaptation. Including all three segments also helps identify reassortment events and improves the accuracy of CCHFV geographic spread.

Seroprevalence of the CCHFV among cattle stood at 10%, with the majority of seropositive animals (n = 34) being over 2 years of age. This indicates a significant correlation between the age of cattle and the increased probability of seropositivity. Every herd examined had at least one animal exposed, illustrating extensive circulation of the CCHFV within the study area. Upon comparing seroprevalence rates in cattle over 2 years old from our study with those from similar age groups in the western region (51%) and the northern region (98%) (Yengoh and Ardö, 2014; Simo Tchetgna et al., 2023), the exposure rate in our study was lower, at 40%. These variations could be explained by several factors such as age, sex, tick infestation rates, vector control strategies, and transhumance. The different ecological context in central Cameroon that favors mainly the circulation of *Rh.* (*B*) *microplus* with respect to *Hyalomma* spp. (the main vector of CCHFV) could, in part, explain this difference in exposure especially with cattle from Northern Cameroon. Age is a recognized risk factor for many vector-borne diseases in animals. Higher tick infestation rates, typical of adult cattle in African settings, and, therefore, an increased likelihood of being in contact with an infected tick over time might translate into an increased probability of CCHFV infection, seroconversion, and, thus, antibody seropositivity. Here, even though older cattle showed higher seroprevalence values than the youngest, it is interesting to note that this exposition increased slightly from the age of one year old showing a local and recent circulation of CCHFV. The risk of introduction of CCHFV in Akonolinga Division by the transhumance of cattle from the north is weak as the proportion of the flow of animals from the northern region to the Center Region varies from 0% to 2% (Organisation Internationale pour les Migrations, 2021). However, another way of introduction may be through the livestock industry, which is largely made up of traditional smallholders and forms a well-organized network, where the animal trade flows between markets (by transfer or movement of animals) have been identified in detail by Motta et al (Motta et al., 2017). The large number of connections and the stability of trade flows between the northern and central regions of Cameroon can possibly constitute a risk of spread of the virus from the northern region to the central region.

In the present study, the higher rates of CCHFV seroprevalence found in cattle compared to small ruminants (goats) were in line with previous studies and could be explained by the fact that cattle tend to be kept longer than small ruminants and then exposed for a longer period to tick bites. Other factors that may be associated with the highest seroprevalence rates in cattle include: animal body size, degree of vascularization, hair (cattle have a larger, well-vascularized body surface with less hair), and husbandry practices (cattle disperse more and travel longer distances than small ruminants that remain in the domestic environment). In agreement with a previous study (Kiwan et al., 2025b), the predominant tick species collected from cattle in the study area during the rainy season were *Rh.* (*B*) *microplus* and *Amblyomma variegatum*. Here, this tick species diversity was confirmed in ticks collected during the dry season. Evidence has demonstrated that the geographic distribution of *Hyalomma* ticks specifically overlaps with human CCHF cases, which is not the case for other tick species, suggesting that the presence of *Hyalomma* ticks may be necessary to support natural circulation of the virus. Interestingly, if more outbreaks of CCHF have been recorded in areas unsuitable for *Hyalomma* ticks, a study in Uganda suggested that *Rhipicephalus* and *Amblyomma* spp. may be responsible for transmitting the virus in the affected areas (Lule et al., 2022). This observation challenges the common assumption that *Hyalomma* tick populations are necessary to maintain CCHFV transmission in nature and suggests the hypothesis that the ecology of this disease may involve other yet-to-be-identified vectors and amplifying hosts across its wide geographical range. While the role of other tick species in CCHFV maintenance and transmission is not well defined, the virus has been detected in several other tick genera (*Amblyomma*, *Rhipicephalus*, *Dermacentor*, and *Ixodes*); however, data on the vector competence of these species are largely missing. CCHFV has been detected in *Amblyomma variegatum* in Ghana, Nigeria, Senegal, and Egypt (Gargili et al., 2017a) and in *Rhipicephalus decolaratus* in Uganda (Wampande et al., 2021).

However, our study has several limitations. Positive samples were not confirmed by neutralization assay to rule out possible cross reactivity with other antigenically related orthonairoviruses. The serological kit used in this study has a specificity of 100% and sensitivity of 98.9% across multiple species; however, the occurrence of unidentified orthonairoviruses cannot be excluded.However this assay has yet been tested in the human population (Sas et al., 2018) and confirmed by field studies (Negredo et al., 2021; Kiwan et al., 2025a). Another limitation includes the small size of the amplified L segment fragment (150 nt), certainly due to the high Ct values (37.79 and 42.62) obtained. This fragment corresponds to a very limited portion of the complete L segment (~12.1 kb). Although this segment appears to be consistent with the S segment, it remains difficult to draw definitive conclusions regarding the specific viral strain identified. Further research is needed to obtain complete genome sequences of CCHFV, as their absence constitutes a major gap in our understanding of the virus’ genetic diversity in this region. The absence of virus detection in ticks collected from cattle could be due to the no collection of *Hyalomma* spp. the main vector of CCHFV. The absence of *Hyalomma* spp. in this agro-ecological zone, characterized by a humid forest–savanna mosaic, has already been observed during the same time period (between April and August) by Silatsa et al (Silatsa et al., 2019). This absence could be related to seasonal conditions that are less conducive to the presence of this species, unlike dry savannah areas where it is found abundant. It may also result from a possible competitive interaction with the invasive species *Rhipicephalus* (*B*) *microplus*, which was abundantly collected in this study (Silatsa et al., 2019). Finally, we have not sampled the wild fauna, especially small mammals, such as hares and hedgehogs. Their role in CCHFV ecology is significant, as population surges (Ergönül, 2006).

## 5 Conclusion

This study provides the first confirmed evidence of CCHFV exposure in humans in Cameroon, together with seroprevalence data in livestock and ticks from the Center Region. The human seroprevalence of 1.9% highlights prior exposure, whereas the detection of viral RNA in two individuals suggests active CCHFV circulation. Notably, these cases occurred in villages where goats also showed seropositivity, reinforcing the zoonotic transmission risk. Phylogenetic analysis of the partial L segment linked the virus to the African III genotype, consistent with strains from Nigeria, Senegal, and Sudan, indicating regional viral circulation. In livestock, cattle exhibited significantly higher seroprevalence than goats, with age being a critical risk factor. Despite the absence of *Hyalomma* spp.—the primary CCHFV vector—ticks such as *Rhipicephalus* (*B*.) *microplus* and *Amblyomma variegatum* dominated, raising questions about their potential roles in virus transmission. The study underlines the value of a One Health approach, integrating human, animal, and environmental data to unravel CCHFV ecology. These findings emphasize the need for expanded surveillance in understudied agroecological zones, complete viral genome sequencing, and investigations into alternative transmission pathways. Further research should explore wild reservoirs, vector competence of local tick species, and the impact of transhumance on viral spread.

## Data Availability

The original contributions presented in the study are publicly available at accession numbers PV112929 for the sample 506-3 and PV112930 for the sample 202-3. This data can be found here: https://www.ncbi.nlm.nih.gov/genbank/.

## References

[B1] Abudurexiti A. Adkins S. Alioto D. Alkhovsky S. V. Avšič-Županc T. Ballinger M. J.. (2019). Taxonomy of the order bunyavirales: update 2019. Arch. Virol. 164, 1949−65. doi: 10.1007/s00705-019-04253-6 31065850 PMC6641860

[B2] Al-Abri S. S. Al Abaidani I. Fazlalipour M. Mostafavi E. Leblebicioglu H. Pshenichnaya N.. (2017). Current status of Crimean-Congo haemorrhagic fever in the World Health Organization Eastern Mediterranean Region: issues, challenges, and future directions. Int. J. Infect. Dis. 58, 82−89. doi: 10.1016/j.ijid.2017.02.018 28259724 PMC7110796

[B3] Awa D. N. Adakal H. Luogbou N. D. D. Wachong K. H. Leinyuy I. Achukwi M. D. (2015). Cattle ticks in Cameroon: Is Rhipicephalus (Boophilus) microplus absent in Cameroon and the Central African region? Ticks Tick-borne Dis. 6, 117−22. doi: 10.1016/j.ttbdis.2014.10.005 25575435

[B4] Ayalew T. Hailu Y. Kumsa B. (2014). Ixodid ticks infesting cattle in three agroecological zones in central oromia: species composition, seasonal variation, and control practices. Comp. Clin. Pathol. 23, 1103−10. doi: 10.1007/s00580-013-1748-y

[B5] Balinandi S. von Brömssen C. Tumusiime A. Kyondo J. Kwon H. Monteil V. M.. (2021). Serological and molecular study of Crimean-Congo hemorrhagic fever virus in cattle from selected districts in Uganda. J. virological Methods 290, 114075. doi: 10.1016/j.jviromet.2021.114075 33515661

[B6] Belobo J. T. E. Kenmoe S. Kengne-Nde C. Emoh C. P. D. Bowo-Ngandji A. Tchatchouang S.. (2021). Worldwide epidemiology of Crimean-Congo Hemorrhagic Fever Virus in humans, ticks and other animal species, a systematic review and meta-analysis. PLoS Negl. Trop. Dis. 15, e0009299. doi: 10.1371/journal.pntd.0009299 33886556 PMC8096040

[B7] Bente D. A. Forrester N. L. Watts D. M. McAuley A. J. Whitehouse C. A. Bray M. (2013). Crimean-Congo hemorrhagic fever: History, epidemiology, pathogenesis, clinical syndrome and genetic diversity. Antiviral Res. 100, 159−89. doi: 10.1016/j.antiviral.2013.07.006 23906741

[B8] Bruijn M. Amadou A. Doksala E. L. Sangaré B. (2016). Mobile pastoralists in Central and West Africa: between conflict, mobile telephony and (im)mobility. Rev. Scientifique Technique- Office Int. Des. Epizooties Future pastoralism 35, 649−57. doi: 10.20506/rst.35.2.2546 27917963

[B9] Donets M. A. Chumakov M. P. Korolev M. B. Rubin S. G. (1977). Physicochemical characteristics, morphology and morphogenesis of virions of the causative agent of Crimean hemorrhagic fever. Intervirology 8, 294–308. doi: 10.1159/000148904 18425

[B10] Ergönül Önder. (2006). Crimean-Congo haemorrhagic fever. Lancet Infect. Dis. 6, 203−14. doi: 10.1016/S1473-3099(06)70435-2 16554245 PMC7185836

[B11] European Virus Archive Global (EVAg). (2020). “Extraction and real-time RT-PCR control (ArRNA MS2) for whole blood and serum samples,” in Standard Operating Procedure. Available at: https://www.european-virus-archive.com/detection-kit/extraction-and-real-time-rt-pcr-control-arrna-ms2-whole-blood-and-serum-samples (Accessed April 14, 2025).

[B12] First Response^®^ Malaria Antigen P.Falciparum (HRP2) Card Test. (2025). (s. d. Premier Medical Corporation Private Limited). Available at: https://www.premiermedcorp.com/product/first-response-malaria-antigen-pfalciparum-hrp2-card-test (Accessed February 5, 2025).

[B13] Formenty P. Schnepf G. Gonzalez-Martin F. Bi Z. (2007). “International surveillance and control of crimean-congo hemorrhagic fever outbreaks,” in Crimean-Congo Hemorrhagic Fever: A Global Perspective. Eds. Ergonul O. Whitehouse C. A. (Springer Netherlands, 295–303 Dordrecht). doi: 10.1007/978-1-4020-6106-6_22

[B14] Gargili A. Estrada-Peña A. Spengler J. R. Lukashev A. Nuttall P. A. Bente D. A. (2017a). The role of ticks in the maintenance and transmission of Crimean-Congo hemorrhagic fever virus: A review of published field and laboratory studies. Antiviral Res. 144, 93−119. doi: 10.1016/j.antiviral.2017.05.010 28579441 PMC6047067

[B15] Gonzalez J. P. Josse R. Johnson E. D. Merlin M. Georges A. J. Abandja J.. (1989). Antibody prevalence against haemorrhagic fever viruses in randomized representative central African populations. Res. Virol. 140, 319–331. doi: 10.1016/S0923-2516(89)80112-8 2505350

[B16] González Gordon L. Bessell P. R. Nkongho E. F. Ngwa V. N. Tanya V. N. Sander M.. (2022). Seroepidemiology of Crimean-Congo Haemorrhagic Fever among cattle in Cameroon: Implications from a One Health perspective. PloS Negl. Trop. Dis. 16, e0010217. doi: 10.1371/journal.pntd.0010217 35312678 PMC8936485

[B17] Grard G. Drexler J. F. Fair J. Muyembe J.-J. Wolfe N. D. Drosten C.. (2011). Re-emergence of Crimean-Congo hemorrhagic fever virus in central Africa. PloS Negl. Trop. Dis. 5, e1350. doi: 10.1371/journal.pntd.0001350 22022629 PMC3191127

[B18] Hufty André. (2001). Introduction à la climatologie: le rayonnement et la température, l’atmosphère, l’eau, le climat et l’activité humaine (France: Presses Université Laval).

[B19] Kaya S. Elaldi N. Kubar A. Gursoy N. Yilmaz M. Karakus G.. (2014). Sequential determination of serum viral titers, virus-specific IgG antibodies, and TNF-alpha, IL-6, IL-10, and IFN-gamma levels in patients with Crimean-Congo hemorrhagic fever. BMC Infect. Dis. 14, 416. doi: 10.1186/1471-2334-14-416 25066751 PMC4133611

[B20] Kiwan P. Gasparine M. Decarreaux D. Capai L. Masse S. Korva M.. (2025a). Serological evaluation of Crimean-Congo Hemorrhagic fever in humans with High-Risk professional exposure and in residual sera collected in 2022–2023 across Corsica (France). One Health 20, 101020. doi: 10.1016/j.onehlt.2025.101020 40230584 PMC11995033

[B21] Kiwan P. Lopez E. Gasparine M. Piorkowski G. Colmant A. Paguem A.. (2025b). First detection and molecular characterization of Jingmen tick virus with a high occurrence in Rhipicephalus (Boophilus) microplus collected from livestock in Cameroon (2024). Parasites Vectors 18, 41. doi: 10.1186/s13071-025-06670-w 39910662 PMC11796043

[B22] Land Cover 2019 (Raster 100 m), Global, Yearly – Version 3. (2025). (s. d. Consulté le 5 février). Available at: https://land.copernicus.eu/en/products/global-dynamic-land-cover/copernicus-global-land-service-land-cover-100m-collection-3-epoch-2019-globe (Accessed February 5, 2025).

[B23] Lakshminarasimhappa M. C. (2022). Web-based and smart mobile app for data collection: kobo toolbox/kobo collect. J. Indian Library Assoc. Now 57, 72−79. Available at: https://Journal.IlaIndia.Net/ (Accessed February 5, 2025).

[B24] Leblebicioglu H. Sunbul M. Guner R. Bodur H. Bulut C. Duygu F.. (2016). Healthcare-associated Crimean-Congo haemorrhagic fever in Turkey, 2002–2014: a multicentre retrospective cross-sectional study. Clin. Microbiol. Infection 22, 387.e1–387.e4. doi: 10.1016/j.cmi.2015.11.024 PMC5023843 26806137

[B25] Lemeshow S. Robinson D. (1985). Surveys to measure programme coverage and impact: A review of the methodology used by the expanded programme on immunization. World Health Stat Quarterly. Rapport Trimestriel Statistiques Sanitaires Mondiales 38, 65−75. 4002731

[B26] Lule S. A. Gibb R. Kizito D. Nakanjako G. Mutyaba J. Balinandi S.. (2022). Widespread exposure to Crimean-Congo haemorrhagic fever in Uganda might be driven by transmission from Rhipicephalus ticks: Evidence from cross-sectional and modelling studies. J. Infection 85, 683−92. doi: 10.1016/j.jinf.2022.09.016 36152736

[B27] Marie Thérèse M. Pierre S. Abdoulmoumini M. Djaouda M. Rodrigue Simonet P. N. Dickmu S.. (2021). Distribution and seasonal dynamics of tick species infesting cattle in nocturnal and daytime systems of livestock in the far north region, Cameroon. Int. J. Anim. Sci. Technol. 5, 47. doi: 10.11648/j.ijast.20210503.11

[B28] MEGA11: Molecular Evolutionary Genetics Analysis Version 11 | Molecular Biology and Evolution | Oxford Academic. (2025). (s. d. Consulté le 5 février). Available at: https://academic.oup.com/mbe/article/38/7/3022/6248099 (Accessed February 5, 2025). 10.1093/molbev/msab120 PMC8233496 33892491

[B29] MINEPIA (2013).Enquête pastorale annuelle 2012. Available online at: www.minepia.cm (Accessed February 5, 2025).

[B30] Molua E. L. (2006). Climatic trends in Cameroon: implications for agricultural management. Climate Res. 30, 255−62. doi: 10.3354/cr030255

[B31] Motta P. Porphyre T. Handel I. Hamman S. M. Ngu Ngwa V. Tanya V.. (2017). Implications of the cattle trade network in Cameroon for regional disease prevention and control. Sci. Rep. 7, 43932. doi: 10.1038/srep43932 28266589 PMC5339720

[B32] Ndengué J. De M. Texier G. Landier J. De Gavelle E. Marchi J. Kamgang L. R.. (2019). Adapting light trap to catch household insects in central Cameroon: A pilot study. Annales la Société Entomologique France (N.S.) 55, 383−94. doi: 10.1080/00379271.2019.1652684

[B33] Negredo A. de la Calle-Prieto F. Palencia-Herrejón E. Mora-Rillo M. Astray-Mochales J. Sánchez-Seco MaríaP.. (2017). Autochthonous Crimean–Congo hemorrhagic fever in Spain. New Engl. J. Med. 377, 154−61. doi: 10.1056/NEJMoa1615162 28700843

[B34] Negredo A. Sánchez-Ledesma M. Llorente F. Pérez-Olmeda M. Belhassen-García M. González-Calle D.. (2021). Retrospective identification of early autochthonous case of Crimean-Congo hemorrhagic fever, Spain, 2013. Emerging Infect. Dis. 27 (6), 1754–1756. doi: 10.3201/eid2706.204643 PMC8153886 34013861

[B35] Ninove L. Nougairede A. Gazin C. Thirion L. Delogu I. Zandotti C.. (2011). RNA and DNA bacteriophages as molecular diagnosis controls in clinical virology: a comprehensive study of more than 45,000 routine PCR tests. PLoS One 6, e16142. doi: 10.1371/journal.pone.0016142 21347398 PMC3036576

[B36] Organisation Internationale pour les Migrations (2021). Matrice de Suivi des Déplacements (DTM). Available online at: https://dtm.iom.int/sites/g/files/tmzbdl1461/files/reports/OIM_CMR_TTT_Dashboard-Comptage_2_mars-mai2021_VF.pdf?iframe=true (Accessed February 5, 2025).

[B37] Sadeuh-Mba S. A. Wansi G. M. Y. Demanou M. Gessain A. Njouom R. (2018). Serological evidence of rift valley fever Phlebovirus and Crimean-Congo hemorrhagic fever orthonairovirus infections among pygmies in the east region of Cameroon. Virol. J. 15, 63. doi: 10.1186/s12985-018-0977-8 29625611 PMC5889602

[B38] Sado Yousseu F. Tchetgna H. S. Kamgang B. Djonabaye D. McCall P. J. Ndip Ndip R.. (2022). Infestation rates, seasonal distribution, and genetic diversity of ixodid ticks from livestock of various origins in two markets of Yaoundé, Cameroon. Med. Veterinary Entomology 36, 283−300. doi: 10.1111/mve.12589 35656818

[B39] Sahak M. N. Arifi F. Saeedzai S. A. (2019). Descriptive epidemiology of Crimean-Congo Hemorrhagic Fever (CCHF) in Afghanistan: Reported cases to National Surveillance System, 2016–2018. Int. J. Infect. Dis. 88, 135−40. doi: 10.1016/j.ijid.2019.08.016 31442628 PMC6853159

[B40] Sánchez-Seco M. Sierra M. Estrada-Peña A. Valcárcel F. Molina R. de Arellano E.. (2022). Widespread detection of multiple strains of crimean-Congo hemorrhagic fever virus in Ticks, Spain. Emerging Infect. Dis. 28, 394–402. doi: 10.3201/eid2802.211308 PMC8798670 35076008

[B41] Sas M. A.. (2018). A novel double-antigen sandwich ELISA for the species-independent detection of Crimean-Congo hemorrhagic fever virus-specific antibodies. Antiviral Res. 151, 24–26. doi: 10.1016/j.antiviral.2018.01.006 29330092

[B42] Silatsa B. A. Kuiate J.-R. Njiokou F. Simo G. Feussom J.-M. K. Tunrayo A.. (2019). A countrywide molecular survey leads to a seminal identification of the invasive cattle tick Rhipicephalus (Boophilus) microplus in Cameroon, a decade after it was reported in Cote d’Ivoire. Ticks Tick-borne Dis. 10, 585–593. doi: 10.1016/j.ttbdis.2019.02.002 30765191 PMC6446184

[B43] Silatsa B. A. Simo G. Githaka N.. (2019). A comprehensive survey of the prevalence and spatial distribution of ticks infesting cattle in different agro-ecological zones of Cameroon. Parasites Vectors 12, 489. doi: 10.1186/s13071-019-3738-7 31623642 PMC6796472

[B44] Simo F. B. N. Teagho U. C. S. Atako S. M. Lontsi B. T. Owona B. V. A. Demanou M.. (2024). Crimean Congo hemorrhagic fever virus exposure among febrile patients, cattle herders, and cattle herds in Cameroon. Acta Tropica 260, 107432. doi: 10.1016/j.actatropica.2024.107432 39427694

[B45] Simo Tchetgna H. Yousseu F. S. Cosset F.-L. de Freitas N. B. Kamgang B. McCall P. J.. (2023). Molecular and serological evidence of crimean-congo hemorrhagic fever orthonairovirus prevalence in livestock and ticks in Cameroon. Front. Cell. Infection Microbiol. 13. doi: 10.3389/fcimb.2023.1132495 PMC10086150 37056704

[B46] Spengler J. R. Bergeron É. Rollin P. E. (2016a). Seroepidemiological studies of Crimean-Congo hemorrhagic fever virus in domestic and wild animals. PLoS Negl. Trop. Dis. 10, e0004210. doi: 10.1371/journal.pntd.0004210 26741652 PMC4704823

[B47] Spengler J. R. Estrada-Peña A. Garrison A. R. Schmaljohn C. Spiropoulou C. F. Bergeron É.. (2016b). A chronological review of experimental infection studies of the role of wild animals and livestock in the maintenance and transmission of Crimean-Congo hemorrhagic fever virus. Antiviral Res. 135, 31−47. doi: 10.1016/j.antiviral.2016.09.013 27713073 PMC5102700

[B48] Walker A. R. Bouattour A. Camicas J.-L. Estrada-Péna A. Horak I. G. Latif A. A.. (2003). “Ticks of domestic animals in africa: A guide to identification of species,” in Bioscience Reports (Univeristy of Edinburgh, Edinburgh Scotland, UK).

[B49] Wampande E. M. Waiswa P. Allen D. J. Hewson R. Frost S. D.W. Stubbs S. C.B. (2021). Phylogenetic characterization of Crimean-Congo hemorrhagic fever virus detected in African blue ticks feeding on cattle in a Ugandan abattoir. Microorganisms 9, 438. doi: 10.3390/microorganisms9020438 33672497 PMC7923759

[B50] Weidmann M. Avsic-Zupanc T. Bino S. Bouloy M. Burt F. Chinikar S.. (2016). Biosafety standards for working with Crimean-Congo hemorrhagic fever virus. J. Gen. Virol. 97, 2799–2808. doi: 10.1099/jgv.0.000610 27667586

[B51] Yengoh G. T. Ardö J. (2014). Crop yield gaps in Cameroon. AMBIO 43, 175–190. doi: 10.1007/s13280-013-0428-0 23925855 PMC3906480

