# Supplementary material for: First report of Crimean-Congo hemorrhagic fever virus exposure in human and livestock populations, Center Region, Cameroon
Source: Front Cell Infect Microbiol. 2025 Jun 9;15:1578518. doi: 10.3389/fcimb.2025.1578518 (PMC12183574; doi:10.3389/fcimb.2025.1578518)
Supplement: Supplementary file 1 [file Table1.docx]

Supplementary Material


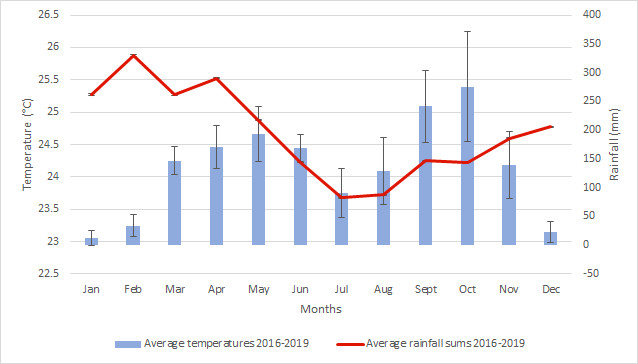


**Supplementary Figure 1.** Monthly average meteorological conditions of Akonolinga district from year 2016 to 2019

**Supplementary Table 1.** Risk factors explored in household respondents

| **Risk factors** | **Overall** |
| --- | --- |
|  | **N = 100***^1^* |
| **Knowledge about tick (Yes)** | **93 (93%)** |
| **Tick-bite one time life** |  |
| Yes | 33 (33%) |
| No | 60 (61%) |
| I don't know | 7 (7%) |
| **Symptoms after tick-bites** |  |
| Yes | 4 (12%) |
| No | 16 (48%) |
| I don't know | 13 (40) |
| **Tick-bite 12 months** |  |
| Yes | 9 (27%) |
| No | 22 (67%) |
| I don't know | 2 (6%) |
| **Tick-bite frequency 12 months** |  |
| 1-5 | 7 (75%) |
| 6-10 | 1 (13%) |
| >10 | 1 (13%) |
| **Crushing tick with bare hands** |  |
| Yes | 67 (69%) |
| No | 33 (31%) |
| **Ever ate engorged ticks** |  |
| Yes | 3 (3%) |
| No | 97 (97%) |
| **Slaughterhouse in households** | 91 (91%) |

*^1^* n (%)

| **Supplementary Table 2.** Overall seroprevalence and village-level seroprevalence of CCHFV in human population. | | | | |
| --- | --- | --- | --- | --- |
|  |  |  |  |  |
| **Village** | **N** | **n** | **%** | **IC 95%** |
| Akanan | 27 | 2 | 7.4 | 2.06-23.37 |
| Efoulan Peuhl | 47 | 1 | 2.13 | 0.38-11.11 |
| Ekougou | 43 | 2 | 4.65 | 1.28-15.46 |
| Mebang | 118 | 2 | 2.15 | 0.59-7.51 |
| Mebolo Assi | 118 | 2 | 2.15 | 0.59-7.51 |
| All individuals included | 465 | 9 | **1.9** | **1.02%-3.64%** |

**Supplementary Table 3.** Characteristics individuals associated with anti-CCHFV antibodies.

| **Characteristic** | **CCHFV_IgG negatif** | **CCHFV_IgG positif** | **Overall** | **P-value2** |
| --- | --- | --- | --- | --- |
|  | N = 456*1* | N = 9*1* | N = 465*1* |  |
| **Sex** |  |  |  | 0.08 |
| Female | 245 (54%) | 2 (22%) | 247 (53%) |  |
| Male | 211 (46%) | 7 (78%) | 218 (47%) |  |
| **Age** | 23 (11, 46) | 42 (23, 66) | 23 (11, 46) | 0.10 |
| **Age_groups** |  |  |  | 0.2 |
| 5-15 | 175 (38%) | 2 (22%) | 177 (38%) |  |
| 16-30 | 100 (22%) | 1 (11%) | 101 (22%) |  |
| 31-45 | 63 (14%) | 2 (22%) | 65 (14%) |  |
| 46-60 | 69 (15%) | 1 (11%) | 70 (15%) |  |
| ≥61 | 49 (11%) | 3 (33%) | 52 (11%) |  |
| *1 n (%); Median (Q1, Q3)* | | | |  |
| 2 Wilcoxon rank sum test; Fisher’s exact test | | | |  |
